# IVF outcomes after hysteroscopic metroplasty in patients with T- shaped uterus

**DOI:** 10.1186/s40738-019-0063-y

**Published:** 2019-12-04

**Authors:** Esra Uyar, Deniz Usal, Belgin Selam, Mehmet Cincik, Tayfun Bagis

**Affiliations:** 1 Acibadem Altunizade Hospital, Unit of ART, Istanbul, Turkey; 2 Department of Obstetrics and Gynecology, Acibadem Mehmet Ali Aydinlar University, School of Medicine; Acibadem Altunizade Hospital, Unit of ART, Altunizade Mah. Tophanelioğlu Cad. Okul Sokak, No:1, Üsküdar, 34662 İstanbul, Turkey; 3 Department of Histology and Embryology, Maltepe University School of Medicine, Istanbul, Turkey

**Keywords:** Hysteroscopic metroplasty, T-shaped uterus, IVF, Live birth rate, Pregnancy rate

## Abstract

**Background:**

T- shaped uterus may be associated with infertility and adverse pregnancy outcomes. Hysteroscopic metroplasty may improve the reproductivity for these cases. To our knowledge, there is no data in literature about the clinical consequences of in vitro fertilization (IVF) in patients undergoing hysteroscopic metroplasty for T-shaped uterus. The principal objective of the current study is to assess the impact of hysteroscopic metroplasty for T-shaped uterus on the reproductive outcomes of IVF.

**Methods:**

IVF outcomes of 74 patients who underwent hysteroscopic metroplasty for T- shaped uterus and 148 patients without any uterine abnormalities and with diagnosis of unexplained infertility (control group) were retrospectively analyzed.

**Results:**

Patients in metroplasty and control groups were comparable with respect to age, BMI, partner’s age and duration of infertility. Number of patients with a history of pregnancy beyond 20 weeks of gestation was significantly lower in the metroplasty group (4.1% vs 18.2%; *p* < 0.05). Number of previous unsuccessful cycles and percentage of patients with ≥3 unsuccessful IVF cycles (35.1% vs 17.6%; *p* < 0.05) were significantly higher in the metroplasty group. There were no significant differences in the reproductive outcomes such as the pregnancy rate, clinical pregnancy or live birth rate between the metroplasty and control groups. There were non-significant trends for higher rates of miscarriage (18.8% vs 8%, *p* > 0.05) and biochemical pregnancy (20.0% vs 10.7%, *p* > 0.05) in the metroplasty group compared to the control group.

**Conclusions:**

Reproductive results of the IVF cycles after hysteroscopic correction of T-shaped uterus were comparable to those of the patients without any uterine abnormalities and with diagnosis of unexplained infertility. Hysteroscopic metroplasty may contribute to improved IVF outcomes in patients with T-shaped uterus.

## Précis

Hysteroscopic correction of T-shaped uterus may be considered in patients with infertility, recurrent abortions or recurrent IV failure.

## Introduction

Mullerian uterine anomalies may be associated with recurrent pregnancy loss, infertility, obstetric complications. Their incidence range between 3.4 and 8.0% in infertile women and 12.6–18.2% in women with recurrent pregnancy loss [1–3].T-shaped uterus is a rare Mullerian anomaly characterized by abnormal shape of the lateral walls in the uterine cavity. T-shaped uterus is most commonly observed in patients with in-utero exposure to diethylstilbestrol (DES). Although DES is no longer administered, T-shaped uterus is still observed in women. Possible etiologies are congenital müllerian malformations or acquired defects due to intrauterine adhesions by the uterine infections or instrumentation [4].

European Society of Human Reproduction and Embryology/European Society for Gynaecological Endoscopy (ESHRE/ESGE) working group of experts described a new classification system for the uterine anomalies [5]. Class UI incorporates all cases with an abnormal shape of lateral wall in the uterine cavity including T-shaped uterus and tubular-shaped/infantilis uteri. Congenital uterine anomalies may be associated with infertility and pregnancy loss as they interfere with normal implantation and placentation [6–12]. Uterine septum and T-shaped uterus may result in functional narrowing of the uterine cavity longitudinally and these abnormalities may lead to defective endometrial receptivity negatively affecting the fertility potential and reproductive performance [12].

One of the first reports about the effects of Müllerian anomalies on in vitro fertilization (IVF) outcomes was conducted by Attia, et al. [13]. The authors analyzed their IVF patients for Mullerian anomalies and compared them with normal women. Some of these anomalies were diagnosed as “DES related anomalies” at the published time (2001), today actually we know that they are T-shaped uteruses. Among 22 patients, their pregnancy rate was 0% whereas it was 24,8% in the control group of 819 patients.

Although the data for the effects of hysteroscopic correction of this anomaly on reproductive outcome is still limited in the literature, gratifying results have been reported following wedge metroplasty in women with T-shaped uterus and recurrent abortions. In one study, the term delivery rate was 10-fold higher after surgical intervention [14].

Considering these data, hysteroscopic metroplasty has been performed in our clinic, before IVF treatment in patients with T-shaped uterus, especially those with additional factors such as prolonged infertility, recurrent abortion, recurrent implantation failure, since 2011.

To our knowledge, there is limited data in literature about the clinical outcomes of IVF in patients undergoing hysteroscopic metroplasty for T-shaped uterus. The objective of the current study is to assess the impact of hysteroscopic metroplasty for T-shaped uterus on the reproductive outcomes of patients in IVF treatment. We compared their IVF data with those of the patients without any uterine abnormalities.

## Materials and methods

Medical records of 101 patients who underwent hysteroscopic correction for T-shaped uterus at Acibadem Kadıköy and Altunizade Hospital, Unit of ART between 2011 and 2016 were analyzed. Data were abstracted from the medical charts of the patients and the missing data about outcome measures were completed through interview of the patients by phone. T-shaped uterus was defined as the narrow uterine cavity due to thickened lateral walls and was diagnosed by hysterosalpingography (HSG) and/or three-dimensional (3D) transvaginal ultrasound [5]. We could not get access to medical records of 11 out of 101 patients. 9 patients got pregnant spontaneously after operation. 7 patients were excluded from the study because treatment cycles were cancelled due to poor ovarian reserve or response. As a result, IVF outcome of 74 out of 101 patients who underwent hysteroscopic correction for T-shaped uterus were compared with 148 patients without any uterine abnormalities. We decided to select patients with good prognosis for the control group. Severe male factor, endometriosis, tubal factor infertility (as a reason of possible undiagnosed hydrosalphinx) or women with diminished ovarian reserve were excluded for this reason. We selected aged matched, unexplained infertility patients who underwent the same treatment protocol in the same years between 2011 and 2016. Demographic variables, characteristics and pregnancy outcomes of IVF cycles were obtained from the hospital records.

The study protocol was approved by the institutional review board of Acibadem Mehmet Ali Aydınlar University, School of Medicine (ATADEK-2018/14). Written informed consent was obtained from each patient for the use of their data in future studies.

### Hysteroscopic Metroplasty Technique

Hysteroscopy was scheduled in the early follicular phase of the menstrual cycle as described before [12]. The operation was performed under general anesthesia in lithotomy position. Mannitol %5 solution (Resectisol, Eczacıbaşı-Baxter, Turkey) was used for distention of the uterine cavity. The pressure of the distension medium was always under the monitorized mean arterial pressure of the patient. The resection was performed using a monopolar hook needle (Resectoscope 26F, Ref:260505 L; optical lens 4 mm, Ref. 26105BA; monopolar hook, Ref. 26050G; Karl Storz, Tuttlingen, Germany). The lateral sidewalls in T-shaped uterus were incised through the tubal ostia to the cervix till a triangular and symmetric cavity was achieved. Combined estrogen and progesterone (estradiol valerate 2 mg for 11 days and estradiol valerate 2 mg plus norgestrel 0,5 mg for 10 days, Cyclo-Progynova; Bayer, Turkey) were administered in the following cycle.

Only the first embryo transfer cycles after operation were analyzed in the study. The metroplasty and control groups were further subdivided into fresh embryo transfer and frozen-thawed embryo transfer (FET) groups to compare the statistical analysis of the cycle parameters.

### Fresh cycle

Fifty-one patients in the metroplasty group and 102 patients in the control group underwent fresh IVF cycle. Pituitary downregulation was employed either by using daily GnRHa leuprolide acetate (Lucrin 5 mg/ml, Abbott, Spain) started in late luteal phase of pre-treatment cycle or by daily GnRH antagonist cetrorelix acetate (Cetrotide, Baxter Oncology GmbH) started by 5th day of the treatment and they were continued until the day of ovulation trigger. Baseline ultrasound scanning was performed on the cycle days 2 or 3. Gonadotropin injections were started if cysts > 2 cm were not observed and the daily dosage ranged between 150 and 300 IU at the physician’s discretion. Ultrasound monitoring continued until the trigger criteria were met with three follicles having a maximum diameter > 17 mm. Oocyte maturation was induced with hCG 10,000 U (Choriomon, IBSA, Italy) in the agonist cycles and with 5000 U hCG (Choriomon, IBSA, Italy) plus 0.2 mg triptorelin acetate (Gonapeptyl, Ferring GmbH Liel, Germany) in the antagonist cycles. Transvaginal ultrasound-guided oocyte retrieval was performed 35–36 h after the hCG injection. Oocyte pickup was performed with a 17-gauge needle for the oocyte retrieval under sedation. The oocyte-corona complexes were denuded, and ICSI was performed after 2 h of incubation. Our clinical policy is to use ICSI routinely in all patients. 1 or 2 embryos were transferred on day 3 or day 5. The embryo transfer policy depends on the number and quality of embryos developed. All patients had the luteal support with estradiol hemihydrate 7,8 mg /3 days (Climara Forte 7.8 mg Flaster, Schering, Germany) and 90 mg of progesterone intravaginally (Crinone 8% gel, Merck) daily, started 1 day after oocyte retrieval. Estradiol was stopped on the day of pregnancy test and progesterone was continued until the negative pregnancy test or until 9 weeks of gestation, in case of pregnancy.

### FET cycles

After the exclusion of ovarian cyst by ultrasonography on the second day of menstruation, patients were started on oral estradiol hemihydrate (Estrofem, Novo Nordisk, Denmark) at a daily dosage of 4 mg for 7 days and 6 mg for 6 days. Serial ultrasound examinations were performed on the 7th and 13th days of treatment. Transvaginal progesterone (Progestan, Kocak Pharma, Turkey) was started on day 14 at a dose of 600 mg/day, embryo transfer was scheduled depending on the cleavega stage of embryos. Both estradiol and progesterone were continued for further 8 weeks, if the pregnancy test result was positive.

A quantitative pregnancy test was performed 10 days after the embryo transfer; a level > 5 IU/L implied a positive test. Implantation rate was calculated as the number of gestational sacs observed divided by the number of embryos transferred. Clinical pregnancy was defined as the observation of a gestational sac on ultrasound scan. Biochemical pregnancy was defined as a positive pregnancy test which subsequently did not rise appropriately or without a gestational sac on the ultrasound scan. Miscarriage was defined as loss of a clinical pregnancy prior to the 20th gestational week. Ongoing pregnancy was a viable pregnancy if the pregnancy continued beyond 8 weeks of gestation. Live birth was defined as delivery after 20 weeks of gestation with at least one alive baby.

Statistical analysis was performed using SPSS version 16.0 (IBM, Armonk, NY, USA). Normally distributed metric variables were tested by the Student-t test for independent samples and the results were expressed as mean ± standard deviation. Non-normally distributed metric variables were analyzed by the Mann-Whitney U test and results were expressed as median (lower quartile-upper quartile). Pearson chi-square test and Fisher’s exact test were used to analyze categorical variables. The results were expressed as percentages (n). All tests were two tailed with a confidence level of 95% (*p* < 0.05).

## Results

Patients in the metroplasty and the control groups were comparable with respect to age, body mass index (BMI), partner’s age and duration of infertility (Table 1). Antral follicle count was lower in the metroplasty group (12.0 ± 5.6 vs 14.2 ± 5.7, *p* < 0.05) (Table 1). Additional infertility factors were detected in 36 patients out of 74 in group 1 (48.6%) including tubal factor (*n* = 3), male factor (*n* = 18), moderate to severe endometriosis (*n* = 7) and two or more infertility factors (*n* = 8).

**Table 1:** Demographic and clinical characteristics of metroplasty and control groups

	Metroplasty (*n* = 74)	Control (*n* = 148)	*p*
Age	34.8 ± 4.5	34.2 ± 4.4	0.36
BMI	22.4 (20.0–26.0)	23.3 (21.1–25.7)	0.21
AFC	12.0 ± 5.6	14.2 ± 5.7	0.005
Partner’s age	36.2 ± 4.8	37.3 ± 5.3	0.12
Duration of infertility	3 (1–7.3)	3 (2–5.8)	0.65

Data are expressed as median (lower quartile-upper quartile) or mean ± SD as appropriate

History of previous pregnancy and IVF trials are demonstrated in Table 2. Only 4.1% (3/74) of patients undergoing metroplasty delivered beyond 20 weeks of gestation compared to 18.2% (20/148) in the control group (*p* < 0.05) (Table 2). Previous miscarriage rate was higher in the metroplasty group, but it was not statistically significant (25.7% vs 16.9%, *p* > 0.05). Also the number of patients with recurrent miscarriages were higher in the metroplasty group, although it did not reach clinical significance (17.6% vs 10.8%, *p* > 0.05) Both number of previous unsuccessful ART cycles and number of patients with ≥3 unsuccessful IVF cycles were significantly higher in the metroplasty group (Table 2).

**Table 2:** Comparison of previous pregnancies and IVF trials between metroplasty and control groups

	Metroplasty (n = 74)	Control (n = 148)	*p*
Patients with ≥1 parity	4.1 (3/74)	18.2 (20/148)	0.004
Patients with pregnancy loss	25.7 (19/74)	16.9 (25/148)	0.12
Patients with ≥2 pregnancy loss	17.6 (13/74)	10.8 (16/148)	0.16
Patients with ≥3 failed IVF cycle	35.1 (26/74)	17.6 (26/148)	0.04

*IVF* In vitro fertilization

Data are expressed as median (lower quartile-upper quartile) or percentages (n) as appropriate

Three patients in the metroplasty group and 6 patients in the control group underwent long GnRH agonist protocol. Forty-eight patients in the metroplasty group and 96 patients in the control group underwent GnRH antagonist protocol. Patients using GnRH agonist and antagonist protocols were combined to form the fresh embryo transfer groups. Estradiol levels, total gonodotropin dose, number of oocytes retrieved, number of MII oocytes, number of embryos transferred and day of the embryo transfer were comparable between both groups among the fresh embryo transferred patients (Table 3). Endometrial thickness was statistically lower in the metroplasty group (9.1 ± 2.0 vs 10.8 ± 2.4, *p* < 0.05) (Table 3). Twenty-three patients in the metroplasty and 46 patients in the control group underwent FET cycle. Cycle parameters were similar among both groups (Table 4).

**Table 3:** Cycle parameters of fresh embryo transfer patients

	Metroplasty (*n* = 51)	Control (*n* = 102)	*p*
Endometrial thickness (mm)	9.1 ± 2.0	10.9 ± 2.2	< 0.001
Estradiol on day of hCG (pg/mL)	1377 (1066–1952)	1270 (962–1827)	0.15
Total gonodotropin dose (IU)	1850 (1400–2400)	1631 (1350–2100)	0.08
Number of oocytes retrieved	10 (6–13)	8 (5–12)	0.08
Number of M2 oocytes	7 (5–10)	6 (4–9)	0.13
Number of embryos transferred	1.9 ± 0.2	1.9 ± 0.4	0.86
Day of embryo transfer			0.14
Day 2 or 3 embryo transfer	58.8 (30/51)	70.6 (72/102)	
Day 5 embryo transfer	41.2 (21/51)	29.4 (30/102)	

Data are expressed as median (lower quartile-upper quartile), mean ± SD or percentages (n) as appropriate

**Table 4:** Cycle parameters of frozen-thawed embryo transfer patients

	Metroplasty (*n* = 23)	Control (*n* = 46)	*p*
Endometrial thickness (mm)	9.6 ± 2.0	10.4 ± 1.9	0.11
Number of embryos transferred	1.7 ± 0.5	1.6 ± 0.5	0.32
Day of embryo transfer			0.13
Day 2 or 3 embryo transfer	60.9 (14/23)	41.3 (19/46)	
Day 5 embryo transfer	39.1 (9/23)	58.7 (27/46)	

Data are expressed as mean ± SD or percentages (n) as appropriate

Rates of implantation (28.1% vs 35.8%, *p* > 0.05), pregnancy (54.1% vs 56.8%, *p* > 0.05), clinical pregnancy (43.2% vs 50.7%, *p* > 0.05) and live birth (35.1% vs 46.6%, *p* > 0.05) were similar between the metroplasty and the control groups, respectively (Table 5). There were non-significant trends toward higher rates of miscarriage (18.8% vs 8%, *p* > 0.05) and biochemical pregnancy (20.0% vs 10.7%, *p* > 0.05) in the metroplasty group (Table 5). Biochemical pregnancy rate was significantly higher among the fresh embryo transferred patients in the metroplasty group (21.9% vs 6.2%, *p* < 0.05) (Table 5).

**Table 5:** Comparison of reproductive outcomes between metroplasty and control groups

	Fresh Embryo Transfer			Frozen-Thawed Embryo Transfer			Total		
	Metroplasty (*n* = 51)	Control (*n* = 102)	*p*	Metroplasty (*n* = 23)	Control (*n* = 46)	*p*	Metroplaty (*n* = 74)	Control (*n* = 148)	*p*
Implantation rate	30.3 (30/99)	41.1 (81/197)	0.70	22.5 (9/40)	21.6 (16/74)	0.91	28.1 (39/139)	35.8 (97/271)	0.11
Pregnancy rate	62.7 (32/51)	62.7 (64/102)	1.00	34.8 (8/23)	43.5 (20/46)	0.48	54.1 (40/74)	56.8 (84/148)	0.70
Clinical pregnancy rate	49.0 (25/51)	58.8 (60/102)	0.25	30.4 (7/23)	32.6 (15/46)	0.85	43.2 (32/74)	50.7 (75/148)	0.30
Biochemical pregnancy rate	21.9 (7/32)	6.2 (4/64)	0.04^a^	12.5 (1/8)	25.0 (5/20)	0.64^a^	20.0 (8/40)	10.7 (9/84)	0.17
Miscarriage rate	12.0 (3/25)	5.0 (3/60)	0.35^a^	42.9 (3/7)	20.0 (3/15)	0.33^a^	18.8 (6/32)	8.0 (6/75)	0.17^a^
LBR	43.1 (22/51)	55.9 (57/102)	0.21	17.4 (4/23)	26.1 (12/46)	0.55^a^	35.1 (26/74)	46.6 (69/148)	0.10

Data are expressed as percentages (n). *LBR* Live birth rate ^a^ Fisher-Exact test

## Discussion

Etiology of the dysmorphic uterus is unclear except in utero exposure to DES. Binding of DES to estrogen receptors during the 9th week of gestation can promote structural irregularities in the uterus [6, 7]. Most prevalent uterine anomalies are T-shaped uterus and hypoplastic uterus [15]. Although DES is not used anymore, dysmorphic uterus is still observed. It may be congenital or secondary to uterine infections or instrumentation [4]. While we have no data about the prevalence of T-shaped uterus in infertile patients, it is reported to be 0.03% in the general population and 0.4% in patients with recurrent pregnancy loss [11]. We estimate that T-shaped uterus will be diagnosed more frequently than reported, as the practice by 3D ultrasound becomes more widespread in the future.

Most of the available data about T-shaped uterus are from studies about DES-exposure. The risk of infertility in DES-exposed women changes with the type of dysmorphism. The risk is multiplied by 1.49 in cases with T-configuration, by 2.26 in cases with midstriction and by 2.63 if both anomalies are present [16]. In a study comparing IVF results between patients with Mullerian anomalies and normal uterus, the DES-exposed women had the poorest outcome, with no ongoing pregnancies in 22 cycles [13]. Karande et al. reported a significantly lower ongoing pregnancy rate in patients with DES exposure compared to women with tubal factor infertility, despite similar number of oocytes retrieved and embryos transferred [17]. These studies suggest that the major factor limiting success in IVF for DES-exposed women is the uterine factor. Even the pregnancy is achieved, outcome appears often compromised with higher rates of ectopic pregnancies, abortions, premature deliveries and low birth weights [2, 6, 15].

Hysteroscopic metroplasty for correction of T-shaped uterus is first published in the 1990s with a very small number of cases [4, 18]. The aim of the surgery is to create the normal pear shape anatomy of the uterine cavity through incisions in the redundant myometrium of the lateral wall and if necessary of the fundus [19] Different surgical modalities have been identified to correct a T-shaped anomaly including scissors, monopolar hook or bipolar electrode systems with very low complication rates [4, 12, 14, 18, 20–22]. Monopolar hook is used during hysteroscopy in the current study.

Significant improvement in the reproductive outcome of patients with dysmorphic uterus has been reported after hysteroscopic correction of dysmorphic uterus in case series [14, 20, 21, 23, 24]. Surgery is beneficial in patients with primary infertility, recurrent implantation failure and poor obstetric history such as repeated miscarriage and preterm delivery. Şükür, et al., followed patients after hysteroscopic correction of T-shaped uterus and septate uterus. The postoperative reproductive performances of both anomalies were similar [12]. Recently, retrospective multicenter and prospective cohort studies demonstrated improved reproductive outcomes following hysteroscopic treatment of T-shaped uterus [25, 26].

There is lack of data in literature analyzing IVF outcomes in detail, following hysteroscopic correction of T-shaped uterus. Most of the studies focused on the long-term reproductive outcome with spontaneous conception and the design of these studies mostly used patients as their own controls.

We aimed to assess the IVF results of patients after hysteroscopic correction of T-shaped uterus. The best way to show these effects of surgery is undoubtedly randomized controlled trial (RCT). However, this is not very easy in daily practice. So, we have shared our experience in a retrospective manner. The time period was restricted to years 2011-2016 to avoid laboratory differences between years. We thought that severe male factor, endometriosis, tubal factor infertility (as reason of possible undiagnosed hydrosalpinx) or women with diminished ovarian reserve patients could affect the success rates and therefore the control group should be patients with best prognosis. For that reason, we selected young women with unexplained infertility. In order to obtain reliable data and reduce the effect of confounding factors we compared the reproductive outcome of operated T-shaped group with a control group of unexplained infertility without any uterine abnormalities.

When we analyzed the demographic data there were some differences between groups. Nearly 35% of the study group consisted of women with repeated IVF failure (control group, 17,6%) and only 4% had parity ≥1 (control group, 18,2%). The difference between the AFC is statistically important but clinically not. As it’s shown, 12 and 14,2 antral follicles do not mean that these women are poor responder patients and the number of oocytes that we retrieved (10 and 8, study and control groups respectively, P:0,08) are not different between the groups. It is not surprising to have lower pregnancy rates in the study group with T-shaped uterus, as they had more failed IVF cycles, and lower parity history. However, the pregnancy rates are nearly the same when compared to the control group. The Live birth rate of 43.1% is very gratifying in the study group which is not statistically different from the very good prognosis patients. We detected similar rates of pregnancy, clinical pregnancy and live birth in both groups. Hysteroscopic metroplasty in patients with T-shaped uterus demonstrated parallel success of IVF treatment compared to patients without any uterine abnormalities.

Ducellier-Azzola, et al. reported a significant reduction of miscarriage rate following enlargement metroplasty [24]. The biochemical pregnancy rate was statistically higher in fresh-embryo transferred patients among hysteroscopically treated T-shaped patients, by subgroup analysis of the current study. Also, statistically non-significant trends for pregnancy loss either as biochemical pregnancy or miscarriage were observed in the metroplasty group. This tendency could be attributed to the expected low miscarriage rates in the control group. The study group had poor prognosis with additional infertility factors, lower mean antral follicle count, higher percentage of patients with history of recurrent IVF failure and recurrent abortions. These factors may affect the higher trend for biochemical pregnancy or miscarriage in the metroplasty group. Hysteroscopic metroplasty may not be adequate to eliminate the increased risk of miscarriage caused by the T-shaped uterus. The shape of the uterus may not be the only factor as it is not precise whether there are endometrial and myometrial, structural or functional differences in these patients. Kupesic and Kurjak postulate that higher and uncoordinated activity of muscular tissue may cause obstetrical complications in patients with vascularized septa [27]. HOXA10, EMX2, TENM1 mRNA and protein expression levels differ significantly in mid-secretory endometrium in infertile women with a Mullerian duct anomaly compared with controls. Abnormal expression of these factors might contribute to the pathogenesis of uterine anomalies and might be a common cause of infertility [28]. Further trials are needed to investigate the reason of miscarriage and the effect of hysteroscopy on pregnancy loss, in patients with T-shaped uterus.

To our knowledge, this might be the first study in literature describing the IVF outcome comprehensively following hysteroscopic metroplasty in patients with T-shaped uterus. We detected similar success rates of IVF following hysteroscopic metroplasty operations in patients with T-shaped uterus compared to patients without any uterine abnormalities. The current data have limitations as this is a retrospective study with a limited sample size. Further studies may use a control group as non-operated patients with T shaped uterus. A prospective controlled study, with a larger sample size, could enlighten whether the operation is beneficial for infertility in these patients. Owing to very low prevalence of T shaped uterus, it is very difficult to make such a study in one center. Multicenter prospective randomized controlled studies would delineate the effect of surgical correction of T-shaped uterus on IVF outcomes.

## Conclusion

These results suggest that hysteroscopic metroplasty may improve IVF outcomes in patients with T-shaped uterus. Hysteroscopic correction of T-shaped uterus may be considered in patients with infertility, recurrent abortions or recurrent IV failure. Considering the limitations of a retrospective study with a limited sample size, multicenter prospective randomized controlled studies would be useful to determine if this data is indeed credible.

## Data Availability

All data generated or analysed during this study is available from the authors upon reasonable request.
